# Characteristics of US Medicare Beneficiaries with Chronic Cough vs. Non-Chronic Cough: 2011–2018

**DOI:** 10.3390/jcm13154549

**Published:** 2024-08-03

**Authors:** Seonkyeong Yang, Shu Huang, Juan M. Hincapie-Castillo, Xuehua Ke, Helen Ding, Mandel R. Sher, Bobby Jones, Debbie L. Wilson, Wei-Hsuan Lo-Ciganic

**Affiliations:** 1 Department of Pharmaceutical Outcomes and Policy, College of Pharmacy, University of Florida, Gainesville, FL 32611, USA; yang.se@ufl.edu (S.Y.); shu.huang@ufl.edu (S.H.); bobby.jones@ufl.edu (B.J.); debbie.wilson@cop.ufl.edu (D.L.W.); 2 Department of Epidemiology, Gillings School of Global Public Health, University of North Carolina at Chapel Hill, Chapel Hill, NC 27599, USA; jhincapie-castillo@unc.edu; 3 Merck & Co., Inc., Rahway, NJ 07065, USAhelen.ding@merck.com (H.D.); 4 Sher Allergy Specialists, Largo, FL 33778, USA; drmrsher@gmail.com; 5 Center for Pharmaceutical Policy and Prescribing, Health Policy Institute, University of Pittsburgh, Pittsburgh, PA 15261, USA; 6 Department of Medicine, School of Medicine, University of Pittsburgh, Pittsburgh, PA 15261, USA; 7 North Florida/South Georgia Veterans Health System Geriatric Research Education and Clinical Center, Gainesville, FL 32608, USA

**Keywords:** chronic cough, Medicare, gabapentinoid, group-based trajectory model, drug utilization, cough medication, antitussive

## Abstract

**Background:** Chronic cough (CC), characterized as a cough lasting >8 weeks, is a common multi-factorial syndrome in the community, especially in older adults. **Methods:** Using a pre-existing algorithm to identify patients with CC within the 2011–2018 Medicare beneficiaries, we examined trends in gabapentinoid use through repeated cross-sectional analyses and identified distinct utilization trajectories using group-based trajectory modeling (GBTM) in a retrospective cohort study. Individuals without CC but with any respiratory conditions related to cough served as a comparator group. **Results:** Among patients with CC, gabapentinoid use increased from 18.6% in 2011 to 24.1% in 2018 (*p* = 0.002), with a similar upward trend observed in the non-CC cohort but with overall lower usage (14.7% to 18.4%; *p* < 0.001). Patients with CC had significantly higher burdens of respiratory and non-respiratory comorbidities, as well as greater healthcare service and medication use compared to the non-CC cohort. The GBTM analyses identified three distinct gabapentinoid utilization trajectories for CC and non-CC patients: no use (77.3% vs. 84.5%), low use (13.9% vs. 10.3%), and high use (8.8% vs. 5.2%). **Conclusions:** Future studies are needed to evaluate the safety and effectiveness of gabapentinoid use in patients with refractory or unexplained CC in real-world settings.

## 1. Introduction

Chronic cough (CC), characterized as a cough lasting longer than 8 weeks, is a common medical condition, particularly among older adults [1,2]. According to a meta-analysis, nearly 10% of the global adult population suffers from CC [3]. The burden of CC increases with age, peaking in the older population [1,4,5,6]. For example, CC prevalence increases from 4 to 6% in 18–29-year-olds to approximately 12% in those aged over 70 years [1]. In addition, an international survey reported that the most common age range for CC patients visiting cough specialist clinics was 60–69 years [7].

CC can manifest in various pulmonary and extrapulmonary conditions such as asthma, chronic obstructive pulmonary disease (COPD), eosinophilic bronchitis, gastroesophageal reflux disease (GERD), and upper airway cough syndrome (UACS) [8]. However, CC often occurs without a known underlying cause (referred to as unexplained CC) and persists despite receiving appropriate cough management (referred to as refractory CC) [9]. In adults, CC is now recognized as a multifactorial syndrome often characterized by cough hypersensitivity, where coughing can be triggered by low-level stimuli, such as underlying medical conditions, environmental factors (e.g., allergens, pollutants), and genetic predispositions [10,11]. The challenges in diagnosing and treating CC, along with its substantial burden, have prompted the recognition of cough hypersensitivity as a distinct clinical entity. Various mechanisms, involving both peripheral and central neural pathways, contribute to this hypersensitivity, which exhibits similarities to chronic pain [12,13,14]. Recent findings indicate that centrally acting neuromodulators commonly used to manage chronic pain, such as gabapentinoids (i.e., gabapentin, pregabalin), and amitriptyline, may offer therapeutic potential for refractory or unexplained CC [14]. The 2016 guideline from the American College of Chest Physicians (CHEST) and the 2020 guideline from the European Respiratory Society (ERS) recommended considering a trial of gabapentin for adults with refractory or unexplained CC [9,15]. However, it is crucial to carefully assess the potential risks of central nervous system (CNS) depression associated with gabapentinoids, which can lead to symptoms like dizziness, drowsiness, somnolence, lethargy, and in severe cases, respiratory depression, particularly among older adults or individuals concurrently using other CNS depressants [16].

Due to the scarcity of literature on gabapentinoid utilization patterns among patients with CC in real-world clinical settings, our study aimed to describe the characteristics of Medicare beneficiaries with CC, analyze the trends in gabapentinoid utilization over time, and identify distinct gabapentinoid utilization trajectories and their determining factors among Medicare beneficiaries with CC.

## 2. Materials and Methods

### 2.1. Data Sources

We used administrative claims data from a nationally representative sample of Medicare beneficiaries from 2011 to 2018, covering approximately 9.6 million beneficiaries. This dataset comprised a 5% national sample of all beneficiaries for the years 2011–2015 and a 15% national sample of fee-for-service beneficiaries for the years 2016–2018. Medicare, the United States (US) government health insurance program, provides coverage for the majority (>93%) of older adults aged 65 and above in US and individuals under 65 with certain disabilities or end-stage renal disease [17,18]. The datasets used in this study included the Medicare master beneficiary summary files, as well as medical claims of inpatient, outpatient, carrier, skilled nursing facility, home health, hospice, and durable medical equipment, and Part D drug event files. Additionally, we linked national provider IDs (NPIs) in medical/Part D claims to the National Plan and Provider Enumeration System (NPPES) file to obtain provider specialty information. Furthermore, we linked the Medicare data with the publicly available Area Health Resource Files (AHRF) to determine if beneficiaries resided in metropolitan or non-metropolitan counties [19]. This study was reviewed and received approval from the University of Florida Institutional Review Board.

### 2.2. Study Design and Cohort

Repeated annual cross-sectional analyses: We conducted repeated annual cross-sectional analyses to examine the trends in gabapentinoid use from 2011 to 2018 among patients with CC. First, we excluded beneficiaries who (1) were aged <18 years (as of June 30th of each measurement year); (2) were non-US residents; (3) had diagnoses of any malignant cancer or respiratory tumors (Table S1); and (4) lacked continuous enrollment in fee-for-service and Part D plans in each calendar year. Our analysis was limited to fee-for-service beneficiaries due to the incomplete capture of healthcare utilization data for beneficiaries enrolled in managed care plans within the dataset. Next, we applied an established algorithm, developed in previous research [20], to ascertain individuals with CC (Figure S1). This algorithm relied on the occurrence of any three clinical cough episodes within a 120-day timeframe, each separated by at least 21 days. These events included either a documented diagnosis of cough (ICD-9-CM: 786.2 or ICD-10-CM: R05) or a filled prescription for CMs, which included: (1) opioid antitussives containing codeine alone or in combination with cold medicines (i.e., antihistamines, expectants, or nasal decongestants), or containing dihydrocodeine or hydrocodone combined with cold medicines; (2) benzonatate; or (3) dextromethorphan, either with or without cold medicines. Given that Medicare Part D plans only reimburse for cough medications when they are used to treat an underlying condition rather than for symptomatic relief [21], the majority of cough episodes in our data comprised medical claims with cough diagnoses. To align with the definition of CC (lasting ≥ 8 weeks), the first and third episodes needed to be at least 56 days apart. A validation study demonstrated this algorithm’s modest sensitivity (15.5%) but high specificity (>99%) [22]. Despite the recognition of gabapentinoids for potential use in refractory or unexplained CC as per the 2016 CHEST and 2020 ERS guidelines [9,15], we chose not to incorporate gabapentinoids into the CC identification algorithm due to their predominant off-label use for chronic pain and other conditions [23].

Retrospective cohort study using group-based trajectory modeling: We performed a retrospective cohort study using group-based trajectory modeling (GBTM) to identify distinct trajectories of gabapentinoid utilization over a 12-month period among patients with CC within Medicare data from 2011 to 2018 (Figure S2). First, we identified eligible beneficiaries by excluding those who (1) were non-US residents and (2) had diagnoses of any malignant cancer or respiratory tumors during the study period. Among eligible beneficiaries, we identified individuals with CC using the same CC algorithm (Figure S1). We defined the date of the first cough episode of three qualifying cough episodes used to determine CC as the index date. When we identified >3 cough episodes during the study period, we used the first 3 qualifying cough episodes to determine the index date. Next, we excluded patients who: (1) were aged <18 years (measured on the index date); (2) had an index date before 1 July 2011, or after 1 January 2018; and (3) lacked continuous enrollment in fee-for-service and Part D plans in the 6-month period before the index date (pre-index period) and the 12-month period after the index date (post-index period).

Comparison group: For both studies, individuals without CC but with any respiratory conditions related to cough served as a comparator group. This group likely experienced acute or sub-acute coughs due to conditions such as acute upper respiratory infections, influenza, bronchitis, pneumonia, cough, and chronic upper respiratory tract diseases (see Table S2 for a detailed list of diagnosis codes) but did not meet the criteria for CC according to the identification algorithm. In the GBTM analysis, the index date for the comparator group was defined as the date of the first cough-related diagnosis.

### 2.3. Outcomes of Interest

In repeated cross-sectional analyses, our primary outcome was gabapentinoid utilization patterns over the 8-year study period. All medications were identified using National Drug Codes (NDCs). In the GBTM analysis, our primary outcome was the patient’s membership in a distinct trajectory of gabapentinoid utilization.

### 2.4. Covariates

We examined socio-demographics and clinical characteristics during the two periods: the 6-month pre-index period and the 12-month post-index period. The socio-demographics included age (at index date), sex, race and ethnicity (Hispanic, non-Hispanic White, non-Hispanic Black, and others), disability status indicating the original reason for Medicare eligibility, receipt of low-income subsidy (LIS) and dual Medicaid eligibility (no LIS or dual eligibility, with only LIS or dual eligibility, and with both LIS and dual eligibility), and rurality of the beneficiary’s county of residence. Measured clinical characteristics comprised the following: (1) comorbid respiratory conditions (e.g., allergic rhinitis, asthma, chronic sinusitis, COPD, pneumonia, pulmonary fibrosis, UACS); (2) comorbid non-respiratory conditions (e.g., GERD, heart failure, musculoskeletal conditions, obesity); (3) the Elixhauser Comorbidity Index (excluding metastatic cancers, solid tumors, and conditions examined individually to avoid collinearity issues [24]); (4) healthcare utilization factors (e.g., any hospitalization, emergency department [ED] visit counts, and outpatient visit counts); (5) receipt of medical procedures (e.g., chest X-ray, laryngoscopy, nasal/sinus endoscopy, spirometry), (6) concomitant medication use (e.g., antidepressants, angiotensin-converting enzyme (ACE) inhibitors, proton pump inhibitors (PPI), corticosteroids). During the 12-month post-index period, we further examined additional clinical characteristics as follows: (1) the number of encounters with respiratory conditions related to cough; (2) the number of gabapentinoid fills; and (3) information regarding specialty visits (i.e., allergist, gastroenterologist, otolaryngologist/head and neck surgeon, pulmonologist, urologist).

### 2.5. Statistical Analysis

In the repeated cross-sectional analyses, we examined the annual gabapentinoid use among CC patients and individuals without CC but with any respiratory conditions related to cough from 2011 to 2018. Next, we tested the significance of trends in the annual gabapentinoid use over time using non-parametric Mann–Kendall trend tests [25].

We employed GBTM to identify the distinct gabapentinoid utilization trajectories over the 12-month post-index period. GBTM, a finite mixture model using maximum likelihood estimation, has the capability to accommodate the dynamic nature of medication use over time in longitudinal data, thereby facilitating the identification of subgroups displaying similar patterns over time [26]. To identify these distinct gabapentinoid utilization trajectories, we first tabulated the monthly count of prescriptions for gabapentinoids over the 12-month post-index period. Next, we modeled the monthly count of gabapentinoid prescriptions using a zero-inflated Poisson distribution in GBTMs with the most flexible functional form of time (e.g., up to the fifth-order polynomial function of time). The optimal number of groups and the best-fitting shape were determined through a comprehensive approach, incorporating the following elements: (1) Bayesian information criterion (BIC), where the largest value indicates the best-fitting model; (2) Nagin’s criteria for evaluating final model adequacy [26,27,28]; and (3) the consideration of clinically interpretable trajectories with a minimum proportion of the cohort (e.g., 5%) for each trajectory. Nagin’s criteria for a well-performing trajectory model consist of several key components: an average posterior probability of ≥0.7 for all groups, an odds of correct classification of ≥5.0 for all groups, and narrow confidence intervals for estimated group membership probabilities [26]. We used traj in STATA 17 (StataCorp LLC, College Stations, TX, USA) for GBTM analysis.

We presented socio-demographics and clinical characteristics measured during pre-index and post-index periods, using percentages for categorical variables and mean and standard deviation (SD) for continuous variables. To compare characteristics between patients with CC and individuals without CC but with any respiratory conditions related to cough, as well as across different gabapentinoid utilization trajectory groups within patients with CC, we employed Student’s *t*-tests for continuous variables and chi-square tests for categorical variables. Multinomial logistic regression was used to identify predictors of gabapentinoid utilization trajectories among patients with CC. To identify pre-index factors associated with these trajectories, we adopted a stepwise variable selection method, with a significance level of 0.05 for entry in the model and 0.01 for staying in it. Additionally, we assessed multicollinearity among pre-index factors using the variance inflation factor. Subsequently, multinomial logistic regression models were executed, incorporating the chosen pre-index factors from the prior steps. Adjusted odds ratios (aORs) with a 95% confidence interval (CI) were then reported. We deemed statistical significance to be present at *p* < 0.05 (two-tailed). All analyses, excluding GBTM, were performed using SAS 9.4 (SAS Institute Inc., Cary, NC, USA).

### 2.6. Subgroup Analyses

We conducted subgroup trend analyses based on age groups (<65 years and ≥65 years).

## 3. Results

### 3.1. Trends in Annual Gabapentinoid Use from Repeated Cross-Sectional Analyses

**Figure 1:**
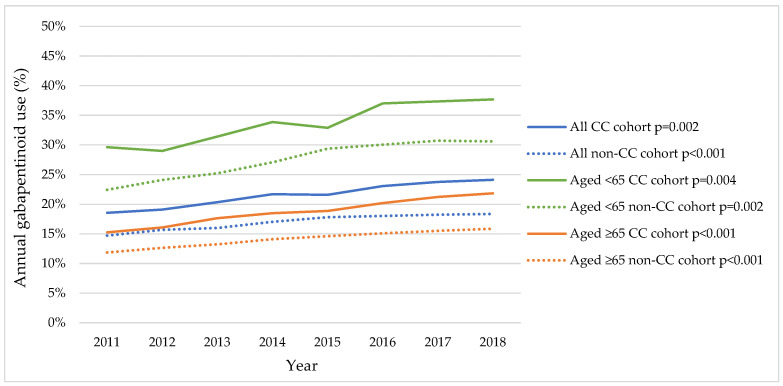
Trends in annual gabapentinoid use in 2011–2018 Medicare data. A *p* < 0.05 indicates significant changes in the trends in annual gabapentinoid use over time.

Among patients with CC, there was a significant increasing trend in gabapentinoid use, rising from 18.6% in 2011 to 24.1% in 2018 (*p* = 0.002) (Table S3 and Figure 1). Similarly, gabapentinoid use increased among individuals without CC but with any respiratory conditions related to cough, albeit with overall low usage compared to patients with CC (14.7% in 2011 to 18.4% in 2018; *p* < 0.001). These upward trends were consistently observed in younger and older adult groups, with younger adults consistently showing higher gabapentinoid usage across the years.

### 3.2. GBTM Analysis: 2011–2018 Medicare Data

#### 3.2.1. Characteristics of Patients with CC and Individuals without CC but with Any Respiratory Conditions Related to Cough

From the national sample of 2011–2018 Medicare data, encompassing 9,645,504 beneficiaries, we identified 39,848 patients with CC (mean age = 71.9 ± 12.5 years, female = 69.0%, non-Hispanic White = 78.4%, disabled = 28.1%) and 831,680 individuals without CC but with any respiratory conditions related to cough (mean age = 70.1 ± 12.7 years, female = 62.4%, non-Hispanic White = 80.5%, disabled = 25.9%) who met all predetermined eligibility criteria (Figure S3 and Table 1). Notably, patients with CC had higher healthcare service utilization (e.g., any hospitalization: 19.0% in the CC cohort vs. 9.7% in the non-CC cohort; *p* < 0.001) and a greater overall prevalence of both respiratory and non-respiratory comorbidities compared to their counterparts without CC during the pre-index period. Among patients with CC, the top five most common respiratory comorbidities were COPD (33.8%), acute upper respiratory tract infections (URTIs) (22.2%), bronchitis (21.8%), asthma (20.0%), and allergic rhinitis (17.6%). In contrast, for the non-CC cohort, the top five respiratory comorbidities were COPD (10.6%), obstructive sleep apnea (7.8%), allergic rhinitis (6.4%), asthma (6.0%), and pulmonary fibrosis (0.8%). The top five non-respiratory comorbidities were consistent across the two groups, although ordered differently; hypertension (71.8% vs. 61.4%) was the most prevalent non-respiratory comorbidity among patients with CC, followed by musculoskeletal conditions (70.6% vs. 57.0%), GERD (34.0% vs. 18.4%), coronary artery disease (29.4% vs. 21.0%), and mood disorders (25.2% vs. 16.2%) and all were significantly different (*p* < 0.001). The comorbidity prevalence during the 12-month post-index period between the two groups remained similar to the pre-index prevalence, albeit slightly higher due to the longer measurement window (Table 1). Procedural and medication use (except ACE inhibitors), including gabapentinoids, was more common among patients with CC compared to their counterparts without CC in the pre-index and post-index periods. During the post-index period, patients with CC had a higher likelihood of visiting an allergist, gastroenterologist, otolaryngologist, pulmonologist, or urologist compared to their counterparts without CC (37.1% vs. 15.9%; *p* < 0.001).

#### 3.2.2. Gabapentinoid Utilization Trajectories

**Figure 2:**
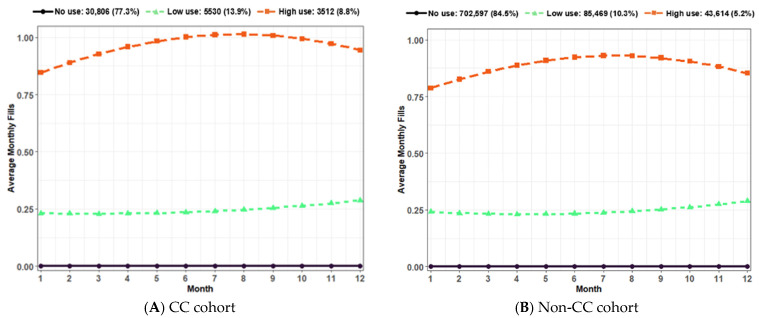
Distinct gabapentinoid utilization trajectories: (**A**) three distinct trajectories identified among patients with CC; (**B**) three distinct trajectories identified among individuals without CC but with any respiratory conditions related to cough. Abbreviation: CC: Chronic Cough.

We identified three distinct gabapentinoid utilization trajectories among patients with CC (Figure 2): (1) no use (*n* = 30,806; 77.3%), (2) low use (*n* = 5530; 13.9%), and (3) high use (*n* = 3512; 8.8%). Over three-quarters of CC patients were not prescribed any gabapentinoids during the 12-month post-index period. Approximately 14% of CC patients consistently received gabapentinoids, averaging 0.25 fills per month. Notably, the high gabapentinoid use group showed nearly consistent monthly refills.

Similarly, we identified three distinct gabapentinoid utilization trajectories among individuals without CC but with any respiratory conditions related to cough: (1) no use (*n* = 702,597; 84.5%), (2) low use (*n* = 85,469; 10.3%), and (3) high use (*n* = 43,614; 5.2%). Over 80% of the cohort did not receive any gabapentinoids during the 12-month post-index period. Within the non-CC cohort, 10% consistently received gabapentinoids, averaging 0.25 monthly fills, while 5% refilled gabapentinoids almost every month.

#### 3.2.3. Characteristics of Patients with CC by Gabapentinoid Utilization Trajectories

The pre-index and post-index characteristics of patients with CC based on their gabapentinoid utilization trajectories are presented in Table S4. Within this CC cohort, gabapentinoid users (age ≥ 65 years: 77.5% in the low use group and 65.4% in the high use group) tended to be younger than non-users (85.4%). Moreover, patients in the low and high gabapentinoid use groups showed higher healthcare utilization, a greater prevalence of overall comorbid conditions, and increased medication use compared to those in the no use group. Notably, specialist visits were generally highest in the low gabapentinoid use group and lowest in the high gabapentinoid use group.

Following stepwise selection and fully adjusted multinomial logistic regression analysis, the pre-index factors associated with gabapentinoid utilization trajectories among patients with CC are presented in Table S5. Compared to the no use group, the pre-index factors found to be significantly positively associated with both the low and high gabapentinoid use groups among patients with CC included the Elixhauser Comorbidity Index, opioid use disorder, the use of gabapentinoids, PPIs, antidepressants, muscle relaxants, non-benzodiazepine hypnotics, and opioid analgesics.

#### 3.2.4. Characteristics of Individuals without CC but with Any Respiratory Conditions Related to Cough by Gabapentinoid Utilization Trajectories

The pre-index and post-index characteristics of individuals without CC but with any respiratory conditions related to cough based on their gabapentinoid utilization trajectories are presented in Table S6. Within this non-CC cohort, gabapentinoid users (age ≥ 65 years: 74.3% in the low use group and 58.8% in the high use group) tended to be younger than non-users (84.1%). In addition, individuals in the low and high gabapentinoid use groups showed higher healthcare utilization, a greater prevalence of overall comorbid conditions, and increased medication use compared to those in the no use group. Notably, specialist visits were generally highest in the low gabapentinoid use group and lowest in the high gabapentinoid use group.

Following stepwise selection and fully adjusted multinomial logistic regression analysis, the pre-index factors associated with gabapentinoid utilization trajectories among the non-CC cohort are presented in Table S7. Due to the large sample size, a greater number of factors were included in the final regression model. Compared to the no use group, strong pre-index factors (aOR ≥ 1.2) found to be significantly positively associated with both the low and high gabapentinoid use groups among the non-CC cohort included disability, musculoskeletal conditions, opioid use disorder, and the use of gabapentinoids, antidepressants, muscle relaxants, non-benzodiazepine hypnotics, and opioid analgesics.

## 4. Discussion

Using a nationally representative sample of Medicare administrative claims data, our study has yielded significant insights into the characteristics and gabapentinoid utilization patterns among patients with CC. First, we identified a substantial disease burden among patients with CC compared to those without CC but with any respiratory conditions related to cough. Patients with CC showed a higher prevalence of both respiratory and non-respiratory comorbidities, as well as increased healthcare utilization and medication use compared to the non-CC cohort. Second, our repeated cross-sectional analyses revealed a statistically significant increasing trend in gabapentinoid use among patients with CC, with similar trends observed across subgroups by age. Third, employing GBTM analyses, we identified three gabapentinoid utilization trajectory groups (no use, low use, and high use) in the CC cohort and non-CC cohort. Across both cohorts, individuals with either low or high gabapentinoid use demonstrated a greater burden of comorbidities and medication use compared to non-users, regardless of CC status.

This study sheds light on the characteristics of patients with CC within clinical settings. Patients with CC showed not only a higher prevalence of respiratory comorbidities, such as COPD, asthma, bronchitis, and allergic rhinitis, but also demonstrated a greater burden of various non-respiratory comorbidities, including GERD, musculoskeletal conditions, coronary artery disease, and anxiety disorders. This higher comorbidity burden in patients with CC led to more frequent use of healthcare services and medications compared to individuals without CC. These findings are consistent with previous studies [4,5,6,7,29]. The presence of multiple comorbidities in patients with CC may contribute to clinical heterogeneity in CC and pose diagnostic and therapeutic challenges. The frequent use of procedures such as chest X-rays, nasal endoscopy, and spirometry among patients with CC suggests diagnostic complexities related to the condition. In addition, multiple comorbidities and potential polypharmacy in older adults with CC raise concerns about high-risk medication use and the prescribing cascade [30]. Therefore, older adults with CC require additional clinical attention.

Gabapentin is recommended for patients with refractory or unexplained CC by recent clinical guidelines [9,15]. We observed an upward trend in gabapentinoid use among patients with CC from 18.6% in 2011 to 24.1% in 2018, which aligns with findings from prior studies. An earlier investigation documented increasing trends in both patients with CC (from 5.3% to 14.4%) and without CC (2.4% to 5.6%) from 2012 to 2021 in Florida [20]. The substantially higher use within both groups identified in our study can largely be attributed to a higher prevalence of comorbid pain conditions among Medicare beneficiaries, primarily consisting of elderly and disabled individuals. Another study, using US nationally representative National Ambulatory Medical Care Survey (NAMCS) data, reported that gabapentinoid use doubled in office-based visits with cough complaints from 1.1% in 2006 to 2.4% in 2018 [9]. Additionally, there are multiple studies in the US reporting a rise in gabapentinoid use in the general adult population [31,32] and the chronic pain population [33]. For example, using US nationally representative Medical Expenditure Panel Survey (MEPS) data, Johansen et al. reported that gabapentinoid use quadrupled from 1.2% in 2002 to 4.7% in 2021 [34]. This upward trend was particularly pronounced among older adults aged ≥65 years and those with multiple comorbidities [35]. Due to multiple off-label indications of gabapentinoids (e.g., pain conditions, mental disorders, and alcohol use disorder) [36] and their widespread use [23], as well as the lack of specific indications in claims data, gabapentinoid use in patients with CC in our study sample may not solely be for treating CC. However, the overall higher prevalence of gabapentinoid use across the study period in the CC cohort compared to the non-CC cohort indicates their potential use for refractory or unexplained CC.

Our GBTM analyses revealed that the majority (~78%) of patients with CC did not use gabapentioids, while the remainder showed chronic usage categorized into high and low use groups. Both groups of gabapentinoid users displayed a higher burden of comorbidities and medication use compared to non-users. The potential risks of abuse and overdose associated with gabapentinoid use [37,38,39,40,41,42] underscore the importance of further investigations into the safety of their usage among patients with CC, given their high comorbidity burden. The highest visits to cough-related specialists were observed in the low gabapentinoid use group, possibly indicating a trial of low-dose gabapentinoid for treating refractory or unexplained CC. Conversely, the lowest visits to cough-related specialists in the high gabapentinoid use group suggest their use for non-cough-related conditions, reducing the need to visit cough-related specialists.

There are several limitations that should be considered in this study. First, there are various potential reasons for the underestimation of CC prevalence, as explained below. (1) We were unable to capture the majority of prescription opioid antitussive, benzonatate, and dextromethorphan use, since they have not been covered by Medicare Part D since 2016, unless used for treating underlying conditions rather than for symptomatic relief [21]. This limitation impacted our ability to identify clinical cough episodes in the CC identification algorithm. (2) The ICD-9-CM/ICD-10-CM codes for cough may not capture all clinical cough episodes. These codes fall under the signs and symptoms section, typically used when signs or symptoms cannot be attributed to an underlying condition. Therefore, it is probable that the CC we captured represents cases of refractory or unexplained CC. However, this limitation may be addressed in the future, as specific ICD-10-CM codes for cough based on the duration (e.g., R05.1: acute cough; R05.2: subacute cough; R05.3: chronic cough) became available 1 October 2021. (3) We were unable to capture over-the-counter dextromethorphan use. To conclude, there is a strong likelihood that we substantially underestimated the CC prevalence among Medicare beneficiaries. The CC patients identified in our study, though, are likely to represent patients with definite CC who require more medical attention. Second, there is a lack of specific indications for gabapentinoid prescriptions, so we were unable to differentiate whether these medications were used for CC or other medical conditions. Third, the monthly counts of gabapentinoid fills, without accounting for days of supply, might have misclassified individuals with longer days of supply as belonging to the low use group. Of note, in 2018, the average duration of gabapentin prescriptions per Medicare beneficiary per year was approximately 28 days, representing a 41% increase compared to 2013 [43]. Lastly, our findings’ generalizability needs to be carefully applied to individuals enrolled in commercial insurance plans or Medicaid, because our analysis was confined exclusively to fee-for-service Medicare beneficiaries.

## 5. Conclusions

Among Medicare beneficiaries, patients with CC had greater comorbidities, medication use, and increased healthcare utilization compared to individuals without CC but with other respiratory conditions related to cough. There was a significant increasing trend in gabapentinoid use among patients with CC. Although the majority of these patients did not use gabapentinoids, approximately 22% used them chronically. Given the abuse potential of gabapentinoids and the high comorbidity burden of patients with CC, further studies are needed to evaluate the safety of their use in this population.

## Figures and Tables

**Table 1:** Patient characteristics of patients with CC and individuals without CC but with any respiratory conditions related to cough: 2011–2018 Medicare data.

Characteristic ^a^	Pre-Index Period ^b^			Post-index Period ^c^		
	CC Cohort	Non-CC Cohort	*p*-Value	CC Cohort	Non-CC Cohort	*p*-Value
N	39,848	831,680		39,848	831,680	
Demographics, %						
Age in years, mean (SD)	71.9 (12.5)	70.1 (12.7)	<0.001	71.9 (12.5)	70.1 (12.7)	<0.001
Age ≥ 65 years	82.5	81.7	<0.001	82.5	81.7	<0.001
Female	69.0	62.4	<0.001	69.0	62.4	<0.001
Race/ethnicity			<0.001			<0.001
Hispanic	8.0	6.7		8.0	6.7	
Non-Hispanic White	78.4	80.5		78.4	80.5	
Non-Hispanic Black	8.6	8.0		8.6	8.0	
Others/multiple/unknown	5.1	4.9		5.1	4.9	
Disability	28.1	25.9	<0.001	28.1	25.9	<0.001
LIS and dual Medicaid eligibility			<0.001			<0.001
No LIS or dual eligibility	61.9	68.4		61.9	68.4	
Only LIS or dual eligibility	3.7	5.2		3.7	5.2	
Both LIS and dual eligibility	34.4	26.4		34.4	26.4	
Residency in a metropolitan area	84.2	81.3	<0.001	84.2	81.3	<0.001
Healthcare utilization factors, %						
Any hospitalization	19.0	9.7	<0.001	35.8	23.3	<0.001
Emergency department visits			<0.001			<0.001
0	66.9	79.6		43.7	57.6	
1	6.4	3.7		4.1	4.0	
≥2	26.7	16.7		52.2	39.4	
Outpatient visits			<0.001			<0.001
0	0.7	3.7		0.0	0.0	
1	0.4	1.4		0.0	0.2	
2–4	2.1	5.7		0.0	0.7	
≥5	96.8	89.2		100.0	99.1	
Comorbidity index, mean (SD)						
Elixhauser index ^d^	2.0 (1.9)	1.4 (1.6)	<0.001	3.1 (2.4)	2.1 (2.1)	<0.001
No. of encounters with respiratory conditions related to cough, mean (SD)						
No. visits with acute URTI	n/m	n/m		2.3 (4.7)	1.8 (3.4)	<0.001
No. visits with bronchitis	n/m	n/m		4.1 (8.6)	1.6 (4.5)	<0.001
No. visits with chronic URTD	n/m	n/m		1.8 (5.7)	0.7 (2.9)	<0.001
No. visits with cough	n/m	n/m		0.6 (3.1)	0.1 (1.0)	<0.001
No. visits with influenza	n/m	n/m		0.6 (3.5)	0.3 (2.6)	<0.001
No. visits with pneumonia	n/m	n/m		5.5 (14.0)	2.1 (8.1)	<0.001
No. visits with any respiratory conditions related to cough	n/m	n/m		13.7 (18.7)	6.2 (10.2)	<0.001
Respiratory comorbidities, %						
Acute URTI	22.2	0.0	<0.001	45.0	52.6	<0.001
Allergic rhinitis	17.6	6.4	<0.001	37.8	16.2	<0.001
Asthma	20.0	6.0	<0.001	36.8	12.2	<0.001
Bronchiectasis	2.8	0.4	<0.001	8.2	1.0	<0.001
Bronchitis	21.8	0.0	<0.001	47.6	32.8	<0.001
Chronic URTD	9.8	0.0	<0.001	26.2	15.0	<0.001
COPD	33.8	10.6	<0.001	58.4	28.4	<0.001
Cough	28.8	0.0	<0.001	100.0	47.2	<0.001
Influenza	1.0	0.0	<0.001	6.4	4.6	<0.001
Obstructive sleep apnea	12.8	7.8	<0.001	20.2	11.6	<0.001
Pneumonia	11.6	0.0	<0.001	31.2	14.8	<0.001
Pulmonary fibrosis	3.6	0.8	<0.001	10.0	2.4	<0.001
UACS	2.6	0.4	<0.001	11.6	2.8	<0.001
Non-respiratory comorbidities, %						
Anxiety disorders	23.4	14.8	<0.001	36.0	24.0	<0.001
Atrial fibrillation	15.0	10.2	<0.001	20.8	14.6	<0.001
Coronary artery disease	29.4	21.0	<0.001	40.0	29.8	<0.001
GERD	34.0	18.4	<0.001	59.2	31.8	<0.001
Heart failure	16.0	7.6	<0.001	26.4	14.2	<0.001
Hypertension	71.8	61.4	<0.001	83.2	75.0	<0.001
Mood disorders	25.2	16.2	<0.001	35.6	24.2	<0.001
Musculoskeletal conditions	70.6	57.0	<0.001	84.8	74.0	<0.001
Non-opioid substance use disorders	5.8	4.6	<0.001	12.2	10.6	<0.001
Obesity	18.8	13.8	<0.001	30.0	23.2	<0.001
Opioid use disorders	2.2	1.4	<0.001	3.6	2.4	<0.001
Other immune disorders	6.6	4.0	<0.001	9.8	6.0	<0.001
Peripheral vascular disease	10.6	6.6	<0.001	16.2	10.8	<0.001
Sleep disturbance	9.6	5.4	<0.001	16.8	10.0	<0.001
Stress incontinence	4.4	2.6	<0.001	7.2	4.4	<0.001
Vomiting	2.4	1.0	<0.001	6.0	2.8	<0.001
Procedures, %						
Allergy radioallergosorbent testing	16.0	10.4	<0.001	36.6	20.8	<0.001
Barium swallow or upper GI imaging	3.2	1.0	<0.001	12.2	3.4	<0.001
Chest CT/MRI/ultrasound	18.6	9.0	<0.001	50.8	23.0	<0.001
Chest X-ray	35.6	15.4	<0.001	82.8	52.0	<0.001
Complete blood count	53.2	44.8	<0.001	88.2	78.6	<0.001
Esophageal endoscopy	5.6	3.4	<0.001	15.2	7.4	<0.001
Laryngoscopy	2.8	0.6	<0.001	14.0	3.4	<0.001
Nasal/sinus endoscopy	10.0	4.6	<0.001	23.4	12.0	<0.001
Sinus X-ray/CT	18.6	12.6	<0.001	39.0	26.4	<0.001
Spirometry	18.6	5.2	<0.001	58.8	19.8	<0.001
Potential cough medication						
Gabapentinoids, %	17.6	12.2	<0.001	22.6	15.6	<0.001
Cardiovascular medications (oral), %						
ACE inhibitors	23.4	25.8	<0.001	22.8	27.2	<0.001
Respiratory medications (oral or inhaled), %						
H1 antihistamines	6.6	3.4	<0.001	10.6	5.8	<0.001
ICS monotherapy	3.6	1.0	<0.001	8.8	2.0	<0.001
ICS/LABA combination	15.0	4.8	<0.001	28.0	7.8	<0.001
LAMA monotherapy	5.8	1.8	<0.001	9.6	2.6	<0.001
Leukotriene modifiers	13.8	4.0	<0.001	25.6	6.8	<0.001
Nasal antihistamines	3.0	0.8	<0.001	8.2	2.2	<0.001
Nasal corticosteroids	17.0	6.8	<0.001	31.8	17.0	<0.001
Nasal SAMA	2.2	0.6	<0.001	6.2	1.8	<0.001
SABA singly inhaled	20.6	6.4	<0.001	40.8	18.6	<0.001
SABA/SAMA combination	3.4	0.8	<0.001	7.6	1.8	<0.001
Gastrointestinal (oral), %						
H2 blockers	10.2	5.8	<0.001	17.6	8.6	<0.001
PPIs	38.4	24.2	<0.001	52.0	29.8	<0.001
Miscellaneous (oral), %						
Corticosteroids	28.4	10.2	<0.001	55.0	30.8	<0.001
Potential respiratory antibiotics	62.0	32.6	<0.001	87.0	78.0	<0.001
Pain medications, psychotherapeutics, others (oral), %						
Antidepressants	40.0	29.4	<0.001	45.6	34.0	<0.001
Antipsychotics	9.6	7.0	<0.001	11.4	8.4	<0.001
Benzodiazepines	23.2	16.4	<0.001	28.0	20.2	<0.001
Muscle relaxants	10.2	7.4	<0.001	15.0	11.2	<0.001
Non-benzodiazepine hypnotics	6.2	4.8	<0.001	7.6	5.8	<0.001
Opioid analgesics	33.8	26.6	<0.001	44.4	36.2	<0.001
Other anxiolytics	2.4	1.6	<0.001	3.4	2.2	<0.001
Other neuromodulators	18.6	12.8	<0.001	21.8	15.4	<0.001
Specialist visits, %						
≥1 visit to allergist	n/m	n/m		6.2	1.2	<0.001
≥1 visit to gastroenterologist	n/m	n/m		1.3	0.8	<0.001
≥1 visit to otolaryngologist/head and neck surgeon	n/m	n/m		15.5	5.9	<0.001
≥1 visit to pulmonologist	n/m	n/m		15.4	2.7	<0.001
≥1 visit to urologist	n/m	n/m		8.7	6.9	<0.001
Visited any specialists specified above	n/m	n/m		37.1	15.9	<0.001
Visited ≥ 2 different specialists specified above	n/m	n/m		30.5	11.8	<0.001
Visited ≥ 3 different specialists specified above	n/m	n/m		25.8	9.2	<0.001
All missing specialty information	n/m	n/m		0.1	0.2	<0.001

Abbreviations: ACE = angiotensin-converting enzyme; CC = chronic cough; COPD = chronic obstructive pulmonary disease; CT = computerized tomography; GERD = gastroesophageal reflux disease; GI = gastrointestinal; H1 = histamine-1 receptor; H2 = histamine-2 receptor; ICS = inhaled corticosteroid; LABA = long-acting beta-agonist; LAMA = long-acting muscarinic-antagonist; LIS = low-income subsidy; MRI = magnetic resonance imaging; n/m = not measured; PPI = proton pump inhibitor; SABA = short-acting beta-agonist; SAMA = short-acting muscarinic-antagonist; SD = standard deviation; UACS = upper airway cough syndrome; URTD = upper respiratory tract disease; URTI = upper respiratory tract infection. ^a^ Characteristics affecting ≥2% of patients with CC and respiratory conditions related to cough. ^b^ Pre-index period is 6 months prior to the index date. ^c^ Post-index period is 12 months after the index date. ^d^ Modified Elixhauser Comorbidity Index was calculated by excluding metastatic cancers, solid tumors, and conditions examined individually.

## Data Availability

The datasets generated or analyzed in this study are not publicly accessible per Centers for Medicare & Medicaid Services (CMS) regulation. Researchers wishing to analyze these datasets must submit a formal application to ResDAC. For more information, please visit their website at https://resdac.org/cms-research-identifiable-request-process-timeline (accessed on 1 August 2024).

## Supplementary Materials

The following supporting information can be downloaded at: https://www.mdpi.com/article/10.3390/jcm13154549/s1, Table S1: ICD-9-CM/ICD-10-CM codes to identify malignant cancer and respiratory tumors, Table S2: Diagnosis codes to identify respiratory conditions related to cough, Table S3: Trends in annual gabapentinoid use in 2011–2018 Medicare data, Table S4: Characteristics of patients with chronic cough by gabapentinoid utilization trajectories: 2011–2018 Medicare data, Table S5: Adjusted odds ratios for pre-index factors associated with gabapentinoid utilization trajectories among patients with chronic cough: 2011–2018 Medicare data, Table S6: Characteristics of individuals without chronic cough but with any respiratory conditions related to cough by gabapentinoid utilization trajectories: 2011–2018 Medicare data, Table S7: Adjusted odds ratios for pre-index factors associated with gabapentinoid utilization trajectories among individuals without chronic cough but with any respiratory conditions related to cough: 2011–2018 Medicare data, Figure S1: Chronic cough identification algorithm, Figure S2: Study design diagram for group-based trajectory modeling (GBTM) analysis, Figure S3: Flowchart for constructing the cohorts for the group-based trajectory modeling (GBTM) analysis: 2011–2018 Medicare data.
