# Supplementary material for: Characteristics of US Medicare Beneficiaries with Chronic Cough vs. Non-Chronic Cough: 2011–2018
Source: J Clin Med. 2024 Aug 3;13(15):4549. doi: 10.3390/jcm13154549 (PMC11312945; doi:10.3390/jcm13154549)
Supplement: Supplementary file 1 [file jcm-13-04549-s001.zip › jcm-3083513-supplementary.pdf]

Supplementary Materials

Table S1. ICD-9-CM/ICD-10-CM codes to identify malignant cancer and respiratory tumors

Table S2. Diagnosis codes to identify respiratory conditions related to cough

Table S3. Trends in annual gabapentinoid use in 2011−2018 Medicare data

Table S4. Characteristics of patients with chronic cough by gabapentinoid utilization trajectories: 2011-2018 Medicare data

Table S5. Adjusted odds ratios for pre-index factors associated with gabapentinoid utilization trajectories among patients with chronic cough: 2011-2018 Medicare data

Table S6. Characteristics of individuals without chronic cough but with any respiratory conditions related to cough by gabapentinoid utilization trajectories: 2011-2018 Medicare data

Table S7. Adjusted odds ratios for pre-index factors associated with gabapentinoid utilization trajectories among individuals without chronic cough but with any respiratory conditions related to cough: 2011-2018 Medicare data

Figure S1. Chronic cough identification algorithm

Figure S2. Study design diagram for group-based trajectory modeling (GBTM) analysis

Figure S3. Flowchart for constructing the cohorts for the group-based trajectory modeling (GBTM) analysis: 2011−2018 Medicare data

Table S1. ICD-9-CM/ICD-10-CM codes to identify malignant cancer and respiratory tumors

Class	ICD-9-CM codes	ICD-10-CM codes
Malignant cancer	140.x 141.x 142.x 143.x 144.x 145.x 146.x 147.x 148.x 149.x 150.x 151.x 152.x 153.x 154.x 155.x 156.x 157.x 158.x 159.x 160.x 161.x 162.x 163.x 164.x 165.x 170.x 171.x 172.x 174.x 175.x 176.x 179.x 180.x 181.x 182.x 183.x 184.x 185.x 186.x 187.x 188.x 189.x 190.x 191.x 192.x 193.x 194.x 195.x 196.x 197.x 198.x 199.x 200.xx 201.xx 202.xx 203.xx 204.xx 205.xx 206.xx 207.xx 208.xx 209.xx	C00.x C01.x C02.x C03.x C04.x C05.x C06.x C07.x C08.x C09.x C10.x C11.x C12.x C13.x C14.x C15.x C16.x C17.x C18.x C19.x C20.x C21.x C22.x C23.x C24.x C25.x C26.x C30.x C31.x C32.x C33.x C34.x C37.x C38.x C39.x C40.x C41.x C43.x C45.x C46.x C47.x C48.x C49.x C4A.0 C4A.10 C4A.11 C4A.12 C4A.20 C4A.21 C4A.22 C4A.30 C4A.31 C4A.39 C4A.4 C4A.51 C4A.52 C4A.59 C4A.60 C4A.61 C4A.62 C4A.70 C4A.71 C4A.72 C4A.8 C4A.9 C50.x C51.x C52.x C53.x C54.x C55.x C56.x C57.x C58.x C60.x C61.x C62.x C63.x C64.1 C64.2 C64.9 C65.1 C65.2 C65.9 C66.1 C66.2 C66.9 C67.0 C67.x C68.x C69.x C70.x C71.x C72.x C73.x C74.x C75.x C76.x C77.x C78.x C79.x C7A.00 C7A.010 C7A.011 C7A.012 C7A.019 C7A.020 C7A.021 C7A.022 C7A.023 C7A.024 C7A.025 C7A.026 C7A.029 C7A.090 C7A.091 C7A.092 C7A.093 C7A.094 C7A.095 C7A.096 C7A.098 C7A.1 C7A.8 C7B.00 C7B.01 C7B.02 C7B.03 C7B.04 C7B.09 C7B.1 C7B.8 C80.0 C80.1 C80.2 C81.x C82.x C83.x C84.x C85.x C86.x C88.x C90.x C91.x C92.x C93.x C94.x C95.x C96.x
Respiratory tumors	210.5 210.6 210.7 210.8 210.9 212.x	D14.x D15.x

Abbreviations: ICD-9/10-CM=International Classification of Diseases, Ninth/Tenth Revision, Clinical Modification

Table S2. Diagnosis codes to identify respiratory conditions related to cough

Conditions	ICD-9-CM codes	ICD-10-CM codes
Acute upper respiratory tract infection	460 461.x 462 464.0x 464.4 465.x	J00 J01.xx J02.x J04.0 J05.0 J06.x
Bronchitis	466.0 490 491.xx	J20.x J40 J41.x J42
Chronic upper respiratory tract diseases	472.x 473.x 476.0	J31.x J32.x J37.0
Cough	786.2	R05
Influenza	487.x 488.xx	J09.xx J10.xx J11.xx
Pneumonia	480.x 481 482.xx 483.x 484.x 485 486	J12.xx J13 J14 J15.xx J16.x J17 J18.x

Abbreviations: ICD-9/10-CM=International Classification of Diseases, Ninth/Tenth Revision, Clinical Modification

Table S3. Trends in annual gabapentinoid use in 2011−2018 Medicare data

Cohort	Individual	2011	2012	2013	2014	2015	2016	2017	2018
**All**									
**CC cohort**	Denominator, n	3,669	4,034	5,443	5,130	5,182	25,954	27,954	28,147
	Gabapentinoid users, n (%)	681 (18.6%)	771 (19.1%)	1,107 (20.3%)	1,113 (21.7%)	1,118 (21.6%)	5,990 (23.1%)	6,642 (23.8%)	6,789 (24.1%)
**Non-CC cohort**	Denominator, n	252,723	262,580	319,789	314,294	306,118	1,276,557	1,334,346	1,301,044
	Gabapentinoid users, n (%)	37,201 (14.7%)	41,160 (15.7%)	51,189 (16.0%)	53,511 (17.0%)	54,557 (17.8%)	229,888 (18.0%)	243,480 (18.2%)	239,013 (18.4%)
**Age <65 years**									
**CC cohort**	Denominator, n	844	945	1,063	1,069	1,000	4,445	4,386	4,040
	Gabapentinoid users, n (%)	250 (29.6%)	274 (29.0%)	334 (31.4%)	362 (33.9%)	329 (32.9%)	1,645 (37.0%)	1,638 (37.3%)	1,523 (37.7%)
**Non-CC cohort**	Denominator, n	68,417	69,400	73,821	70,617	66,633	249,185	239,475	220,210
	Gabapentinoid users, n (%)	15,351 (22.4%)	16,732 (24.1%)	18,619 (25.2%)	19,125 (27.1%)	19,573 (29.4%)	74,852 (30.0%)	73,585 (30.7%)	67,367 (30.6%)
**Age ≥65 years**									
**CC cohort**	Denominator, n	2,825	3,089	4,380	4,061	4,182	21,509	23,568	24,107
	Gabapentinoid users, n (%)	431 (15.3%)	497 (16.1%)	773 (17.6%)	751 (18.5%)	789 (18.9%)	4,345 (20.2%)	5,004 (21.2%)	5,266 (21.8%)
**Non-CC cohort**	Denominator, n	184,306	193,180	245,968	243,677	239,485	1,027,372	1,094,871	1,080,834
	Gabapentinoid users, n (%)	21,850 (11.9%)	24,428 (12.6%)	32,570 (13.2%)	34,386 (14.1%)	34,984 (14.6%)	155,036 (15.1%)	169,895 (15.5%)	171,646 (15.9%)

Abbreviation: CC=Chronic cough

Table S4. Characteristics of patients with chronic cough by gabapentinoid utilization trajectories: 2011-2018 Medicare data

Characteristics[a]	Pre-index period[b]				Post-index period[c]			
	**No use**	**Low use**	**High use**	**p-value**	**No use**	**Low use**	**High use**	**p-value**
**N**	30,783	5,530	3,512		30,783	5,530	3,512	
**Demographics, %**								
Age in years, mean (SD)	72.6 (12.2)	70.1 (12.2)	69.0 (14.3)	<0.001	72.6 (12.2)	70.1 (12.2)	69.0 (14.3)	<0.001
Age ≥65 years	85.4	77.5	65.4	<0.001	85.4	77.5	65.4	<0.001
Female	67.8	73.3	72.4	<0.001	67.8	73.3	72.4	<0.001
Race/ethnicity				<0.001				<0.001
Hispanic	7.7	9.2	8.7		7.7	9.2	8.7	
Non-Hispanic White	79.1	75.3	76.8		79.1	75.3	76.8	
Non-Hispanic Black	7.9	10.8	10.8		7.9	10.8	10.8	
Others/multiple/unknown	5.3	4.7	3.6		5.3	4.7	3.6	
Disability	23.9	35.9	52.0	<0.001	23.9	35.9	52.0	<0.001
LIS & dual Medicaid eligibility				<0.001				<0.001
No LIS or dual eligibility	66.3	58.2	29.2		66.3	58.2	29.2	
Only LIS or dual eligibility	3.4	4.3	4.7		3.4	4.3	4.7	
Both LIS and dual eligibility	30.3	37.5	66.0		30.3	37.5	66.0	
Residency in a metropolitan area	85.1	82.8	78.6		85.1	82.8	78.6	
**Healthcare utilization factors, %**								
Any hospitalization	16.6	23.3	34.3	<0.001	31.8	45.9	54.5	<0.001
Emergency department visits				<0.001				<0.001
0	70.1	60.1	49.4		47.5	33.0	26.8	
1	5.8	7.3	10.4		4.0	4.2	4.6	
≥2	24.1	32.6	40.2		48.5	62.8	68.6	
Outpatient visits				<0.001				0.35
0	0.9	0.2	*		0.0	0.0	0.0	
1	0.5	0.2	*		0.0	0.0	0.0	
2-4	2.5	1.1	0.4		0.0	0.0	0.0	
≥5	96.2	98.4	99.3		100.0	100.0	100.0	
**Comorbidity index, mean (SD)**								

Elixhauser index[d]	1.8 (1.8)	2.4 (2.1)	3.1 (2.2)	<0.001	2.8 (2.3)	3.7 (2.5)	4.4 (2.5)	<0.001
**No. of encounters with respiratory conditions related to cough, mean (SD)**								
No. visits with acute URTI	n/m	n/m	n/m		2.2 (4.6)	2.6 (5.1)	2.5 (5.1)	<0.001
No. visits with bronchitis	n/m	n/m	n/m		3.8 (8.2)	5.1 (9.6)	5.2 (9.8)	<0.001
No. visits with chronic URTD	n/m	n/m	n/m		1.9 (5.8)	2.0 (5.9)	1.2 (4.4)	<0.001
No. visits with cough	n/m	n/m	n/m		0.6 (3.3)	0.5 (2.6)	0.4 (2.5)	<0.001
No. visits with influenza	n/m	n/m	n/m		0.6 (3.3)	0.8 (3.8)	0.8 (3.9)	<0.001
No. visits with pneumonia	n/m	n/m	n/m		4.8 (12.9)	6.6 (16.3)	9.5 (18.2)	<0.001
No. visits with any respiratory conditions related to cough	n/m	n/m	n/m		12.7 (17.7)	16.2 (20.9)	18.3 (22.0)	<0.001
**Respiratory comorbidities, %**								
Acute URTI	21.6	24.4	23.0	<0.001	44.4	48.0	47.0	<0.001
Allergic rhinitis	17.6	18.8	15.8	0.001	38.4	38.8	31.4	<0.001
Asthma	18.6	24.8	25.0	<0.001	35.6	43.2	37.6	<0.001
Bronchiectasis	3.0	2.6	2.4	0.07	8.6	7.8	5.8	<0.001
Bronchitis	20.8	25.0	25.2	<0.001	46.0	52.2	54.2	<0.001
Chronic URTD	9.4	11.4	9.4	<0.001	26.4	28.4	20.0	<0.001
COPD	31.2	38.8	49.8	<0.001	55.8	64.0	72.6	<0.001
Cough	28.2	29.8	32.8	<0.001	100.0	100.0	100.0	n/a
Influenza	1.0	1.2	1.4	0.14	6.0	7.4	8.2	<0.001
Obstructive sleep apnea	11.4	18.2	17.6	<0.001	18.2	27.8	25.8	<0.001
Pneumonia	10.4	12.6	20.4	<0.001	29.2	33.8	45.4	<0.001
Pulmonary fibrosis	3.4	4.0	4.6	<0.001	9.8	11.2	9.4	0.01
UACS	2.8	2.4	2.0	0.01	12.0	11.8	7.0	<0.001
**Non-respiratory comorbidities, %**								
Anxiety disorders	20.4	28.0	42.0	<0.001	32.0	44.4	57.0	<0.001
Atrial fibrillation	14.8	14.6	17.6	<0.001	20.4	21.2	23.6	<0.001
Coronary artery disease	27.8	33.8	37.6	<0.001	38.0	45.4	48.4	<0.001
GERD	31.6	40.4	46.2	<0.001	56.8	67.4	67.6	<0.001
Heart failure	14.2	19.0	27.6	<0.001	23.8	31.4	41.2	<0.001
Hypertension	69.8	77.0	81.4	<0.001	81.8	87.8	89.8	<0.001
Mood disorders	21.4	30.4	50.6	<0.001	30.8	44.0	63.4	<0.001

Musculoskeletal conditions	66.2	83.8	87.2	<0.001	82.0	94.8	95.0	<0.001
Non-opioid substance use disorders	5.2	7.2	8.4	<0.001	11.4	15.2	13.8	<0.001
Obesity	16.6	25.2	28.2	<0.001	26.8	39.0	42.2	<0.001
Opioid use disorders	1.2	4.6	7.8	<0.001	2.2	6.8	12.0	<0.001
Other immune disorders	5.8	9.8	9.8	<0.001	8.4	14.4	13.8	<0.001
Peripheral vascular disease	9.2	13.4	18.8	<0.001	14.2	20.6	27.2	<0.001
Sleep disturbance	8.2	12.6	17.4	<0.001	14.4	22.2	28.2	<0.001
Stress incontinence	4.0	5.6	5.6	<0.001	6.6	9.4	9.6	<0.001
Vomiting	2.2	3.0	4.4	<0.001	5.6	6.6	10.2	<0.001
**Procedures, %**								
Allergy radioallergosorbent testing	15.0	19.8	18.8	<0.001	35.2	35.2	43.0	37.8
Barium swallow or upper GI imaging	3.0	4.0	4.8	<0.001	11.5	11.6	14.8	14.4
Chest CT/MRI/ultrasound	17.0	23.0	25.8	<0.001	49.2	49.2	58.4	52.2
Chest X-ray	33.2	40.4	48.2	<0.001	81.5	81.4	86.0	88.8
Complete blood count	51.8	57.6	58.2	<0.001	87.3	87.4	91.2	91.6
Esophageal endoscopy	5.0	7.0	8.2	<0.001	14.6	14.6	18.4	15.8
Laryngoscopy	2.6	3.4	3.0	0.001	14.0	14.0	16.6	10.6
Nasal/sinus endoscopy	9.6	10.0	12.6	<0.001	23.2	23.2	24.0	24.2
Sinus X-ray/CT	17.6	21.6	24.4	<0.001	37.1	37.2	45.4	45.2
Spirometry	17.4	23.2	21.0	<0.001	58.3	58.4	65.8	52.4
**Potential cough medication**								
Gabapentinoids, %	3.4	51.6	89.0	<0.001	0.0	100.0	100.0	<0.001
**Cardiovascular medications (oral), %**								
ACE inhibitors	22.8	25.0	26.4	<0.001	21.8	24.6	28.2	<0.001
**Respiratory medications (oral or inhaled)**, %								
H1 antihistamines	5.4	9.2	13.2	<0.001	9.0	14.6	19.8	<0.001
ICS monotherapy	3.6	3.8	4.6	0.01	8.4	9.2	10.6	<0.001
ICS/LABA combination	13.6	17.6	22.2	<0.001	26.2	32.2	37.6	<0.001
LAMA monotherapy	5.0	6.6	10.0	<0.001	8.6	11.8	15.6	<0.001
Leukotriene modifiers	13.0	17.0	16.4	<0.001	25.0	29.0	25.6	<0.001
Nasal antihistamines	2.8	3.4	2.6	0.05	8.4	8.6	5.2	<0.001
Nasal corticosteroids	16.0	19.2	22.4	<0.001	30.6	35.4	35.8	<0.001

Nasal SAMA	2.2	2.6	1.8	0.05	6.4	7.0	3.8	<0.001
SABA singly inhaled	18.8	24.4	29.4	<0.001	39.2	45.0	50.2	<0.001
SABA/SAMA combinations	3.0	2.4	9.0	<0.001	6.6	5.8	19.0	<0.001
**Gastrointestinal (oral), %**								
H2 blockers	9.2	12.4	16.4	<0.001	16.4	20.2	22.8	<0.001
PPIs	35.0	46.2	54.4	<0.001	49.0	62.0	63.8	<0.001
**Miscellaneous (oral), %**								
Corticosteroids	26.6	35.0	33.6	<0.001	53.0	63.6	59.6	<0.001
Potential respiratory antibiotics	59.8	67.6	72.8	<0.001	85.6	90.8	92.8	<0.001
**Pain medications, psychotherapeutics, others (oral), %**								
Antidepressants	35.2	50.2	66.6	<0.001	40.4	58.0	72.0	<0.001
Antipsychotics	8.4	9.0	20.8	<0.001	10.0	12.2	23.4	<0.001
Benzodiazepines	20.4	29.2	37.8	<0.001	25.0	35.8	42.8	<0.001
Muscle relaxants	7.0	17.6	26.6	<0.001	11.0	26.0	33.4	<0.001
Non-benzodiazepine hypnotics	5.2	9.4	10.0	<0.001	6.6	11.4	11.0	<0.001
Opioid analgesics	27.2	51.0	65.2	<0.001	37.2	65.8	73.6	<0.001
Other anxiolytics	2.0	2.8	6.2	<0.001	2.6	4.4	8.8	<0.001
Other neuromodulators	16.6	21.0	33.6	<0.001	19.0	25.8	39.2	<0.001
**Specialist visits, %**								
≥1 visit to allergist	n/m	n/m	n/m		6.4	6.8	3.5	<0.001
≥1 visit to gastroenterologist	n/m	n/m	n/m		1.3	1.3	1.2	0.89
≥1 visit to otolaryngologist/head and neck surgeon	n/m	n/m	n/m		15.8	17.8	9.3	<0.001
≥1 visit to pulmonologist	n/m	n/m	n/m		15.6	16.8	12.2	<0.001
≥1 visit to urologist	n/m	n/m	n/m		8.5	10.0	8.2	<0.001
Visited to any specialists specified above	n/m	n/m	n/m		37.4	41.3	28.2	<0.001
Visited to ≥2 different specialists specified above	n/m	n/m	n/m		30.6	34.2	23.8	<0.001
Visited to ≥3 different specialists specified above	n/m	n/m	n/m		25.9	28.8	21.0	<0.001
All missing specialty information	n/m	n/m	n/m		0.1	*	*	0.40

Abbreviations: ACE=angiotensin-converting enzyme; COPD=chronic obstructive pulmonary disease; CT=computerized tomography; GERD=gastroesophageal reflux disease; GI=gastrointestinal; HIPAA=Health Insurance Portability and Accountability Act; H1=histamine-1 receptor; H2=histamine-2 receptor; ICS=inhaled corticosteroid; LABA=long-acting beta-agonist; LAMA=long-acting muscarinic antagonist; LIS=low-income subsidy; MRI=magnetic resonance imaging; n/m=not measured; PPI=proton pump inhibitor; SABA=short-acting beta agonist; SAMA=short-acting muscarinic antagonist; SD=standard deviation; UACS=upper airway cough syndrome; URTD=upper respiratory tract disease; URTI=upper respiratory tract infection.* A percentage from a count of patients less than 11 or used to derive a count of patients less than 11 in other cells is not reportable according to HIPAA Privacy Rule. [a] Characteristics affecting ≥2% of patients with CC and respiratory conditions related to cough. [b] Pre-index period is 6 months prior to the index date. [c] Post-index period is 12 months after the index date. [d] Modified Elixhauser Comorbidity Index was calculated by excluding metastatic cancers, solid tumors, and conditions examined individually.

Table S5. Adjusted odds ratios for pre-index factors associated with gabapentinoid utilization trajectories among patients with chronic cough: 2011-2018 Medicare data

Pre-index factors	Low use aOR (95% CI)	High use aOR (95% CI)
**Demographics**		
Age (each one year increase)[a]	0.99 (0.99-1.00)	1.01 (1.01-1.02)
Female	1.15 (1.07-1.25)	0.96 (0.85-1.07)
Race/ethnicity		
Hispanic	1.27 (1.12-1.44)	0.75 (0.63-0.91)
Non-Hispanic White	Reference	Reference
Non-Hispanic Black	1.14 (1.00-1.29)	0.77 (0.65-0.92)
Others/multiple/unknown	1.20 (1.03-1.41)	0.87 (0.67-1.12)
Disability	1.07 (0.97-1.19)	1.31 (1.14-1.51)
LIS & dual Medicaid eligibility		
No LIS or dual eligibility	Reference	Reference
Only LIS or dual eligibility	1.10 (0.92-1.32)	2.32 (1.81-2.99)
Both LIS and dual eligibility	0.92 (0.84-1.01)	2.65 (2.33-3.01)
Residency in a metropolitan area	0.90 (0.82-0.99)	0.81 (0.71-0.92)
**Comorbidities (Yes vs. No)**		
Elixhauser index (each one unit increase)[a,b]	1.05 (1.03-1.07)	1.09 (1.06-1.12)
Obstructive sleep apnea	1.17 (1.06-1.29)	0.97 (0.85-1.12)
Pneumonia	0.90 (0.80-1.01)	1.12 (0.97-1.29)
Pulmonary fibrosis	1.14 (0.95-1.37)	1.49 (1.15-1.92)
Mood disorder	0.96 (0.88-1.05)	1.28 (1.13-1.45)
Musculoskeletal conditions	1.45 (1.33-1.58)	1.14 (0.99-1.32)
Opioid use disorders	1.73 (1.41-2.13)	1.81 (1.41-2.32)
**Procedure and medication use (Yes vs. No)**		
Spirometry	1.13 (1.03-1.23)	0.89 (0.78-1.01)
Gabapentinoids	23.87 (21.95-25.96)	156.48 (137.82-177.66)
SABA/SAMA combinations	0.58 (0.46-0.73)	1.19 (0.95-1.50)
Proton pump inhibitors	1.09 (1.02-1.18)	1.14 (1.03-1.26)
Oral corticosteroids	1.12 (1.04-1.21)	0.98 (0.87-1.09)
Antidepressants	1.27 (1.17-1.37)	1.50 (1.33-1.68)
Antipsychotics	0.74 (0.65-0.85)	1.17 (1.00-1.37)
Muscle relaxants	1.23 (1.10-1.38)	1.47 (1.28-1.68)
Non-benzodiazepine hypnotics	1.40 (1.23-1.59)	1.31 (1.10-1.57)
Opioid analgesics	1.41 (1.31-1.52)	1.58 (1.42-1.77)

Abbreviations: aOR=adjusted odds ratio; CI=confidence interval; LIS=low-income subsidy; SABA=short-acting beta agonist; SAMA=short-acting muscarinic antagonist. [a] Continuous variable. [b] Modified Elixhauser Comorbidity Index was calculated by excluding metastatic cancers, solid tumors, and conditions examined individually.

Table S6. Characteristics of individuals without chronic cough but with any respiratory conditions related to cough by gabapentinoid utilization trajectories: 2011-2018 Medicare data

Characteristics[a]	Pre-index period[b]				Post-index period[c]			
	No use	Low use	High use	p-value	No use	Low use	High use	p-value
**N**	702,597	85,469	43,614		702,597	85,469	43,614	
**Demographics, %**								
Age in years, mean (SD)	70.5 (12.6)	68.8 (12.5)	66.0 (14.4)	<0.001	70.5 (12.6)	68.8 (12.5)	66.0 (14.4)	<0.001
Age ≥65 years	84.1	74.3	58.8	<0.001	84.1	74.3	58.8	<0.001
Female	61.7	66.2	66.3	<0.001	61.7	66.2	66.3	<0.001
Race/ethnicity				<0.001				<0.001
Hispanic	6.4	8.3	8.1		6.4	8.3	8.1	
Non-Hispanic White	81.1	77.1	77.9		81.1	77.1	77.9	
Non-Hispanic Black	7.6	10.3	10.4		7.6	10.3	10.4	
Others/multiple/unknown	5.0	4.3	3.7		5.0	4.3	3.7	
Disability	22.7	37.2	54.9	<0.001	22.7	37.2	54.9	<0.001
LIS & dual Medicaid eligibility				<0.001				<0.001
No LIS or dual eligibility	71.3	61.0	35.6		71.3	61.0	35.6	
Only LIS or dual eligibility	5.1	5.5	6.5		5.1	5.5	6.5	
Both LIS and dual eligibility	23.6	33.5	57.9		23.6	33.5	57.9	
Residency in a metropolitan area	82.0	80.2	74.4	<0.001	82.0	80.2	74.4	<0.001
**Healthcare utilization factors, %**								
Any hospitalization	8.6	14.4	18.6	<0.001	20.9	35.0	39.5	<0.001
Emergency department visits				<0.001				<0.001
0	81.5	71.6	64.7		60.4	44.5	38.3	
1	3.3	5.0	6.5		3.9	4.4	5.4	
≥2	15.2	23.4	28.8		35.8	51.1	56.3	
Outpatient visits				<0.001				<0.001
0	4.1	1.4	0.9		0.0	0.0	*	
1	1.6	0.7	0.5		0.2	0.0	*	
2-4	6.3	3.1	2.3		0.8	0.1	0.1	
≥5	88.0	94.8	96.3		98.9	99.9	99.9	

**Comorbidity index, mean (SD)**								
Elixhauser index[d]	1.2 (1.5)	1.9 (1.8)	2.2 (1.9)	<0.001	2.0 (2.0)	2.9 (2.3)	3.3 (2.4)	<0.001
**No. of encounters with respiratory conditions related to cough, mean (SD)**								
No. visits with acute URTI	n/m	n/m	n/m		1.8 (3.3)	1.9 (3.7)	1.8 (3.8)	<0.001
No. visits with bronchitis	n/m	n/m	n/m		1.5 (4.3)	2.0 (5.1)	2.1 (5.4)	<0.001
No. visits with chronic URTD	n/m	n/m	n/m		0.6 (2.8)	0.8 (3.2)	0.7 (2.9)	<0.001
No. visits with cough	n/m	n/m	n/m		0.1 (1.0)	0.1 (0.9)	0.1 (0.8)	<0.001
No. visits with influenza	n/m	n/m	n/m		0.3 (2.5)	0.4 (2.8)	0.5 (3.2)	<0.001
No. visits with pneumonia	n/m	n/m	n/m		1.9 (7.7)	2.8 (9.5)	4.1 (11.4)	<0.001
No. visits with any respiratory conditions related to cough	n/m	n/m	n/m		5.9 (9.7)	7.4 (11.7)	8.7 (13.3)	<0.001
**Respiratory comorbidities, %**								
Acute URTI	0.0	0.0	0.0	n/a	53.0	52.0	48.0	<0.001
Allergic rhinitis	6.4	7.2	6.8	<0.001	16.0	17.4	15.8	<0.001
Asthma	5.4	8.6	10.0	<0.001	11.4	16.6	17.4	<0.001
Bronchiectasis	0.4	0.4	0.4	0	1.0	1.2	1.0	0.008
Bronchitis	0.0	0.0	0.0	n/a	32.4	35.8	35.4	<0.001
Chronic URTD	0.0	0.0	0.0	n/a	14.8	16.4	14.2	<0.001
COPD	9.4	15.2	20.4	<0.001	26.6	35.4	42.0	<0.001
Cough	0.0	0.0	0.0	n/a	46.6	49.6	52.0	<0.001
Influenza	0.0	0.0	0.0	n/a	4.4	4.8	5.0	<0.001
Obstructive sleep apnea	7.0	12.4	11.8	<0.001	10.4	18.0	17.4	<0.001
Pneumonia	0.0	0.0	0.0	n/a	13.8	17.8	24.2	<0.001
Pulmonary fibrosis	0.8	1.0	1.0	0.2	2.2	3.0	3.2	<0.001
UACS	0.4	0.4	0.4	0.4	2.8	2.8	2.0	<0.001
**Non-respiratory comorbidities, %**								
Anxiety disorders	13.0	21.2	30.8	<0.001	21.6	34.0	45.0	<0.001
Atrial fibrillation	10.0	11.0	11.2	<0.001	14.2	16.2	16.4	<0.001
Coronary artery disease	20.2	25.6	25.8	<0.001	28.6	36.6	36.4	<0.001
GERD	17.0	25.0	29.2	<0.001	29.6	43.0	46.6	<0.001
Heart failure	6.8	10.6	14.0	<0.001	12.8	19.4	24.0	<0.001
Hypertension	59.6	70.2	71.0	<0.001	73.6	83.0	82.6	<0.001

Mood disorders	14.0	23.4	37.0	<0.001	21.2	35.6	50.8	<0.001
Musculoskeletal conditions	53.2	75.4	80.4	<0.001	70.8	90.6	91.2	<0.001
Non-opioid substance use disorders	4.2	6.2	7.2	<0.001	10.0	13.6	14.2	<0.001
Obesity	12.4	20.6	22.4	<0.001	21.4	33.0	35.4	<0.001
Opioid use disorders	1.0	3.4	6.6	<0.001	1.4	5.8	10.2	<0.001
Other immune disorders	3.6	6.8	7.2	<0.001	5.2	10.4	10.6	<0.001
Peripheral vascular disease	5.6	9.8	13.4	<0.001	9.4	16.8	20.6	<0.001
Sleep disturbance	4.8	8.8	11.0	<0.001	8.6	16.0	18.8	<0.001
Stress incontinence	2.2	3.6	4.0	<0.001	4.0	6.4	6.6	<0.001
Vomiting	1.0	1.6	2.2	<0.001	2.4	4.0	5.0	<0.001
**Procedures, %**								
Allergy radioallergosorbent testing	9.6	14.6	14.8	<0.001	19.4	29.4	28.0	<0.001
Barium swallow or upper GI imaging	1.0	1.6	1.8	<0.001	3.2	4.6	5.4	<0.001
Chest CT/MRI/ultrasound	8.4	12.4	13.2	<0.001	21.4	30.8	31.6	<0.001
Chest X-ray	14.0	20.8	25.0	<0.001	49.8	62.0	66.6	<0.001
Complete blood count	43.6	51.2	53.0	<0.001	77.4	85.4	85.8	<0.001
Esophageal endoscopy	3.0	4.8	5.0	<0.001	6.8	10.8	10.0	<0.001
Laryngoscopy	0.6	1.0	1.0	<0.001	3.2	4.2	4.2	<0.001
Nasal/sinus endoscopy	4.6	5.2	6.2	<0.001	11.8	12.6	13.6	<0.001
Sinus X-ray/CT	12.0	15.0	16.4	<0.001	25.0	33.0	34.4	<0.001
Spirometry	4.8	7.4	8.0	<0.001	18.8	25.4	25.6	<0.001
**Potential cough medication**								
Gabapentinoids, %	2.2	54.8	88.2	<0.001	0.0	100.0	100.0	<0.001
**Cardiovascular medications (oral), %**								
ACE inhibitors	25.2	28.8	30.2	<0.001	26.6	30.8	32.0	<0.001
**Respiratory medications (oral or inhaled)**, %								
H1 antihistamines	2.8	5.8	9.2	<0.001	5.0	9.6	14.0	<0.001
ICS monotherapy	1.0	1.2	1.6	<0.001	1.8	2.4	3.0	<0.001
ICS/LABA combination	4.4	6.2	8.8	<0.001	7.2	10.4	13.6	<0.001
LAMA monotherapy	1.6	2.4	3.4	<0.001	2.4	3.6	5.0	<0.001
Leukotriene modifiers	3.8	5.6	6.4	<0.001	6.4	8.8	9.4	<0.001
Nasal antihistamines	0.6	0.8	0.8	<0.001	2.2	2.4	2.2	<0.001

Nasal corticosteroids	6.4	8.8	10.8	<0.001	16.4	19.8	21.2	<0.001
Nasal SAMA	0.6	0.6	0.6	<0.001	1.8	2.0	1.6	<0.001
SABA singly inhaled	5.6	9.2	13.6	<0.001	17.4	23.8	29.4	<0.001
SABA/SAMA combinations	0.6	0.8	2.0	<0.001	1.6	1.6	5.4	<0.001
**Gastrointestinal (oral), %**								
H2 blockers	5.2	7.6	11.4	<0.001	7.8	11.6	15.6	<0.001
PPIs	22.0	34.4	41.4	<0.001	27.2	42.2	48.2	<0.001
**Miscellaneous (oral), %**								
Corticosteroids	9.2	15.4	15.0	<0.001	29.4	40.4	37.0	<0.001
Potential respiratory antibiotics	31.0	40.4	44.8	<0.001	77.0	83.8	85.2	<0.001
**Pain medications, psychotherapeutics, others (oral), %**								
Antidepressants	26.0	42.8	58.6	<0.001	30.2	50.4	64.6	<0.001
Antipsychotics	6.4	7.2	16.8	<0.001	7.6	9.4	19.6	<0.001
Benzodiazepines	14.6	23.8	32.4	<0.001	18.2	29.2	36.8	<0.001
Muscle relaxants	5.2	16.0	24.8	<0.001	8.4	24.4	31.6	<0.001
Non-benzodiazepine hypnotics	4.2	7.8	9.4	<0.001	5.2	9.6	10.8	<0.001
Opioid analgesics	21.8	48.0	61.8	<0.001	30.8	63.0	70.2	<0.001
Other anxiolytics	1.2	2.4	4.8	<0.001	1.8	3.6	6.6	<0.001
Other neuromodulators	11.4	16.4	27.2	<0.001	13.6	20.8	31.6	<0.001
**Specialist visits, %**								
≥1 visit to allergist	n/m	n/m	n/m		1.3	1.3	0.9	<0.001
≥1 visit to gastroenterologist	n/m	n/m	n/m		0.7	1.0	0.9	<0.001
≥1 visit to otolaryngologist/head and neck surgeon	n/m	n/m	n/m		5.9	6.8	4.9	<0.001
≥1 visit to pulmonologist	n/m	n/m	n/m		2.5	3.6	3.2	<0.001
≥1 visit to urologist	n/m	n/m	n/m		6.7	8.4	7.2	<0.001
Visited to any specialists specified above	n/m	n/m	n/m		15.6	18.8	15.4	<0.001
Visited to ≥2 different specialists specified above	n/m	n/m	n/m		11.5	14.1	11.8	<0.001
Visited to ≥3 different specialists specified above	n/m	n/m	n/m		8.9	11.0	9.7	<0.001

All missing specialty information	n/m	n/m	n/m		0.3	0.1	0.2	<0.001

Abbreviations: ACE=angiotensin-converting enzyme; COPD=chronic obstructive pulmonary disease; CT=computerized tomography; GERD=gastroesophageal reflux disease; GI=gastrointestinal; HIPAA=Health Insurance Portability and Accountability Act; H1=histamine-1 receptor; H2=histamine-2 receptor; ICS=inhaled corticosteroid; LABA=long-acting beta-agonist; LAMA=long-acting muscarinic antagonist; LIS=low-income subsidy; MRI=magnetic resonance imaging; n/m=not measured; PPI=proton pump inhibitor; SABA=short-acting beta agonist; SAMA=short-acting muscarinic antagonist; SD=standard deviation; UACS=upper airway cough syndrome; URTD=upper respiratory tract disease; URTI=upper respiratory tract infection.* A percentage from a count of patients less than 11 or used to derive a count of patients less than 11 in other cells is not reportable according to HIPAA Privacy Rule. [a] Characteristics affecting ≥2% of patients with CC and respiratory conditions related to cough. [b] Pre-index period is 6 months prior to the index date. [c] Post-index period is 12 months after the index date. [d] Modified Elixhauser Comorbidity Index was calculated by excluding metastatic cancers, solid tumors, and conditions examined individually.

Table S7. Adjusted odds ratios for pre-index factors associated with gabapentinoid utilization trajectories among individuals without chronic cough but with any respiratory conditions related to cough: 2011-2018 Medicare data

Pre-index factors	Low use aOR (95% CI)	High use aOR (95% CI)
**Demographics**		
Age (each one year increase)[a]	1.00 (1.00-1.00)	1.01 (1.01-1.01)
Female	1.06 (1.04-1.08)	0.96 (0.93-0.98)
Race/ethnicity		
Hispanic	1.21 (1.17-1.26)	0.80 (0.76-0.84)
Non-Hispanic White	Reference	Reference
Non-Hispanic Black	1.18 (1.14-1.22)	0.86 (0.82-0.91)
Others/multiple/unknown	1.09 (1.04-1.14)	0.85 (0.79-0.91)
Disability	1.30 (1.26-1.33)	1.54 (1.48-1.60)
LIS & dual Medicaid eligibility		
No LIS or dual eligibility	Reference	Reference
Only LIS or dual eligibility	1.13 (1.09-1.18)	2.09 (1.97-2.22)
Both LIS and dual eligibility	0.98 (0.96-1.01)	2.38 (2.30-2.47)
Residency in a metropolitan area	0.96 (0.93-0.98)	0.77 (0.75-0.80)
**Healthcare utilization factors**		
Outpatient visits		
0	0.99 (0.93-1.06)	1.22 (1.07-1.38)
1	0.92 (0.83-1.01)	0.93 (0.78-1.09)
2-4	0.95 (0.91-1.00)	0.92 (0.85-1.00)
≥5	Reference	Reference
**Comorbidities (Yes vs. No)**		
Elixhauser index (each one unit increase)[a,b]	1.07 (1.06-1.08)	1.12 (1.11-1.13)
Allergic rhinitis	0.94 (0.91-0.98)	0.89 (0.84-0.94)
Asthma	1.03 (1.00-1.07)	0.96 (0.91-1.01)
COPD	1.08 (1.05-1.11)	1.09 (1.04-1.13)
Obstructive sleep apnea	1.20 (1.17-1.24)	1.03 (0.98-1.08)
Atrial fibrillation	0.94 (0.91-0.96)	1.02 (0.97-1.06)
Coronary artery disease	0.99 (0.97-1.02)	0.87 (0.84-0.90)
Heart failure	1.00 (0.96-1.03)	1.11 (1.06-1.16)
Hypertension	1.10 (1.07-1.12)	1.05 (1.02-1.09)
Mood disorders	1.00 (0.97-1.02)	1.12 (1.08-1.16)
Musculoskeletal conditions	1.39 (1.36-1.42)	1.29 (1.25-1.34)
Non-opioid substance use disorders	1.15 (1.10-1.20)	1.00 (0.94-1.06)
Obesity	1.11 (1.09-1.14)	1.03 (1.00-1.07)
Opioid use disorders	1.25 (1.18-1.33)	1.50 (1.39-1.61)
Other immune disorders	1.07 (1.03-1.11)	1.05 (1.00-1.12)
Peripheral vascular disease	1.16 (1.12-1.20)	1.38 (1.32-1.44)
Sleep disturbance	1.12 (1.08-1.16)	1.10 (1.05-1.16)
Stress incontinence	1.10 (1.05-1.16)	1.08 (1.00-1.16)

Vomiting	0.86 (0.79-0.93)	0.84 (0.76-0.93)
**Procedure and medication use**		
Allergy radioallergosorbent testing	1.06 (1.03-1.09)	0.97 (0.93-1.01)
Chest CT/MRI/ultrasound	1.04 (1.01-1.07)	0.99 (0.94-1.03)
Chest X-ray	0.96 (0.93-0.98)	0.97 (0.93-1.00)
Complete blood count	0.95 (0.94-0.97)	0.92 (0.90-0.95)
Esophageal endoscopy	1.07 (1.03-1.12)	0.99 (0.92-1.05)
Nasal/sinus endoscopy	0.93 (0.90-0.97)	0.99 (0.94-1.05)
Sinus X-ray/CT	0.95 (0.93-0.98)	0.92 (0.89-0.96)
Spirometry	1.03 (0.99-1.07)	0.95 (0.90-1.00)
Gabapentinoids	38.74 (37.92-39.58)	209.02 (201.97-216.32)
ACE inhibitors	1.03 (1.01-1.05)	1.03 (1.00-1.06)
H1 antihistamines	1.09 (1.05-1.14)	1.13 (1.06-1.19)
ICS monotherapy	0.86 (0.79-0.93)	0.90 (0.80-1.01)
Nasal corticosteroids	1.08 (1.04-1.11)	1.12 (1.07-1.17)
SABA singly inhaled	1.05 (1.01-1.09)	1.13 (1.08-1.19)
SABA/SAMA combination	0.80 (0.72-0.89)	1.23 (1.09-1.39)
H2 blockers	0.98 (0.95-1.02)	1.16 (1.11-1.21)
PPIs	1.17 (1.15-1.20)	1.20 (1.17-1.23)
Corticosteroids	1.08 (1.05-1.11)	0.94 (0.90-0.97)
Potential respiratory antibiotics	1.04 (1.02-1.06)	1.05 (1.02-1.08)
Antidepressants	1.24 (1.22-1.27)	1.47 (1.43-1.52)
Antipsychotics	0.72 (0.70-0.75)	1.08 (1.03-1.13)
Benzodiazepines	1.12 (1.09-1.14)	1.22 (1.18-1.26)
Muscle relaxants	1.30 (1.26-1.34)	1.43 (1.38-1.49)
Non-benzodiazepine hypnotics	1.20 (1.16-1.25)	1.22 (1.16-1.29)
Opioid analgesics	1.47 (1.44-1.50)	1.70 (1.65-1.75)
Other anxiolytics	1.10 (1.03-1.17)	1.30 (1.20-1.41)
Other neuromodulators	0.99 (0.96-1.02)	1.26 (1.22-1.31)

Abbreviations: ACE=angiotensin-converting enzyme; aOR=adjusted odds ratio; CI=confidence interval; COPD=chronic obstructive pulmonary disease; CT=computerized tomography; H1=histamine-1 receptor; H2=histamine-2 receptor; ICS=inhaled corticosteroid; LIS=low-income subsidy; MRI=magnetic resonance imaging; PPI=proton pump inhibitor; SABA=short-acting beta agonist; SAMA=short-acting muscarinic antagonist. [a] Continuous variable. [b] Modified Elixhauser Comorbidity Index was calculated by excluding metastatic cancers, solid tumors, and conditions examined individually.

Figure S1. Chronic cough identification algorithm

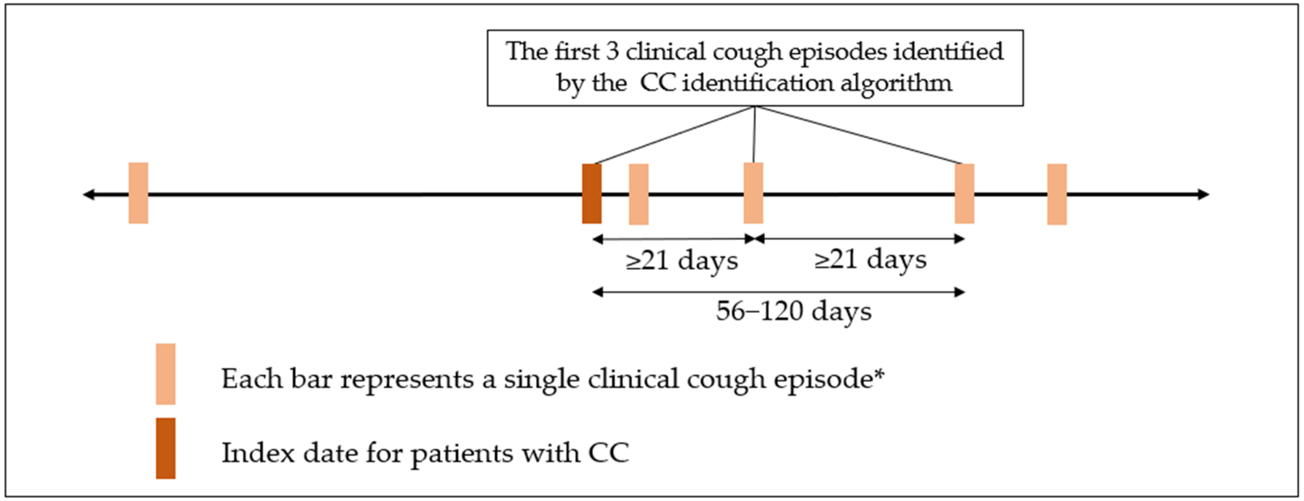

Abbreviations: CC=chronic cough. * A documented diagnosis of cough (ICD-9-CM: 786.2 or ICD-10-CM: R05) or a filled prescription for CMs, including opioid antitussives, benzonatate, or dextromethorphan. To qualify as chronic cough, any two out of three clinical cough episodes within a 120-day timeframe must be at least 3 weeks apart, and the first and third episodes must be separated by at least 56 days.

Figure S2. Study design diagram for group-based trajectory modeling (GBTM) analysis

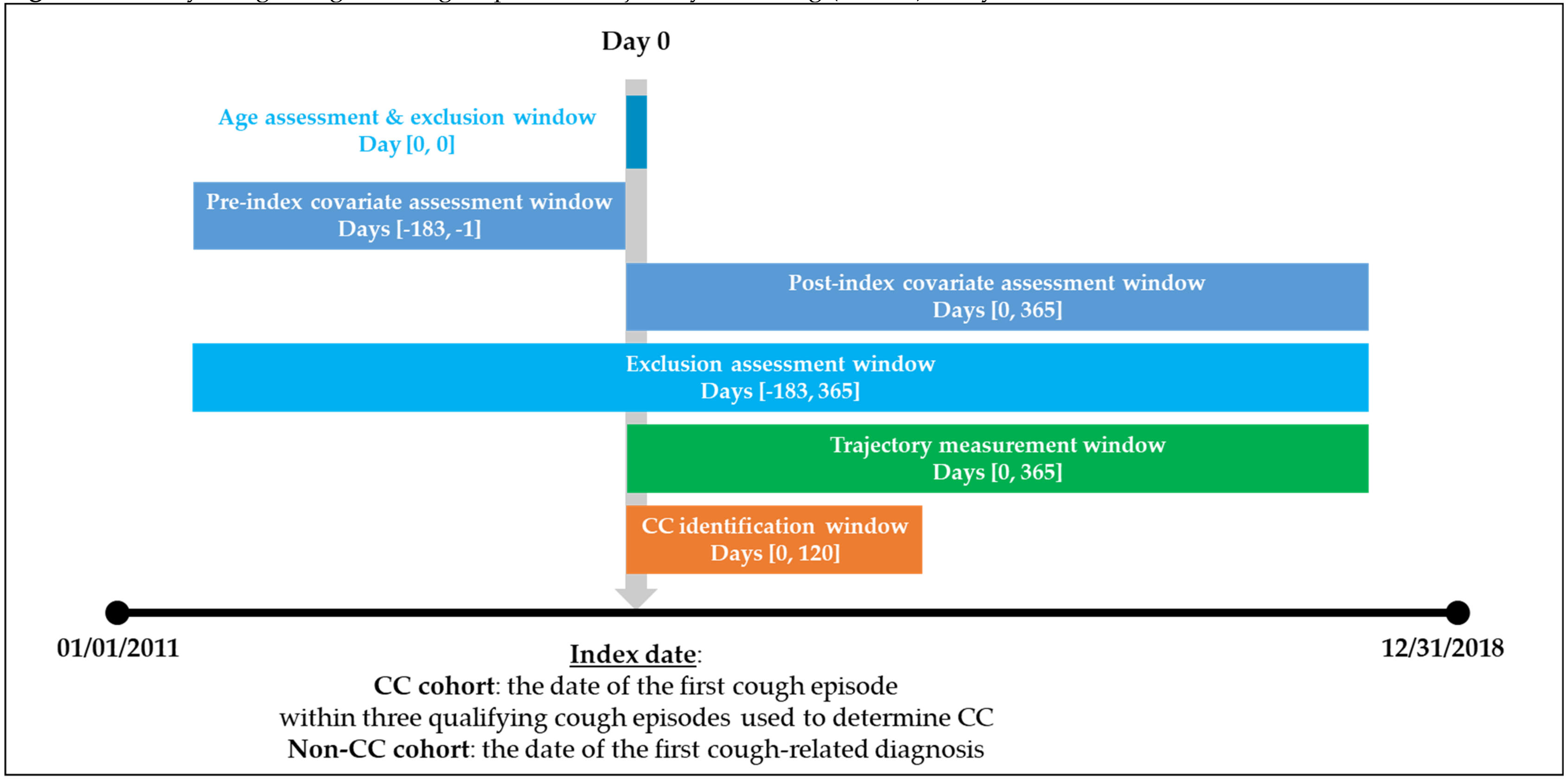

Abbreviations: CC: Chronic Cough.

Figure S3. Flowchart for constructing the cohorts for the group-based trajectory modeling (GBTM) analysis: 2011−2018 Medicare data

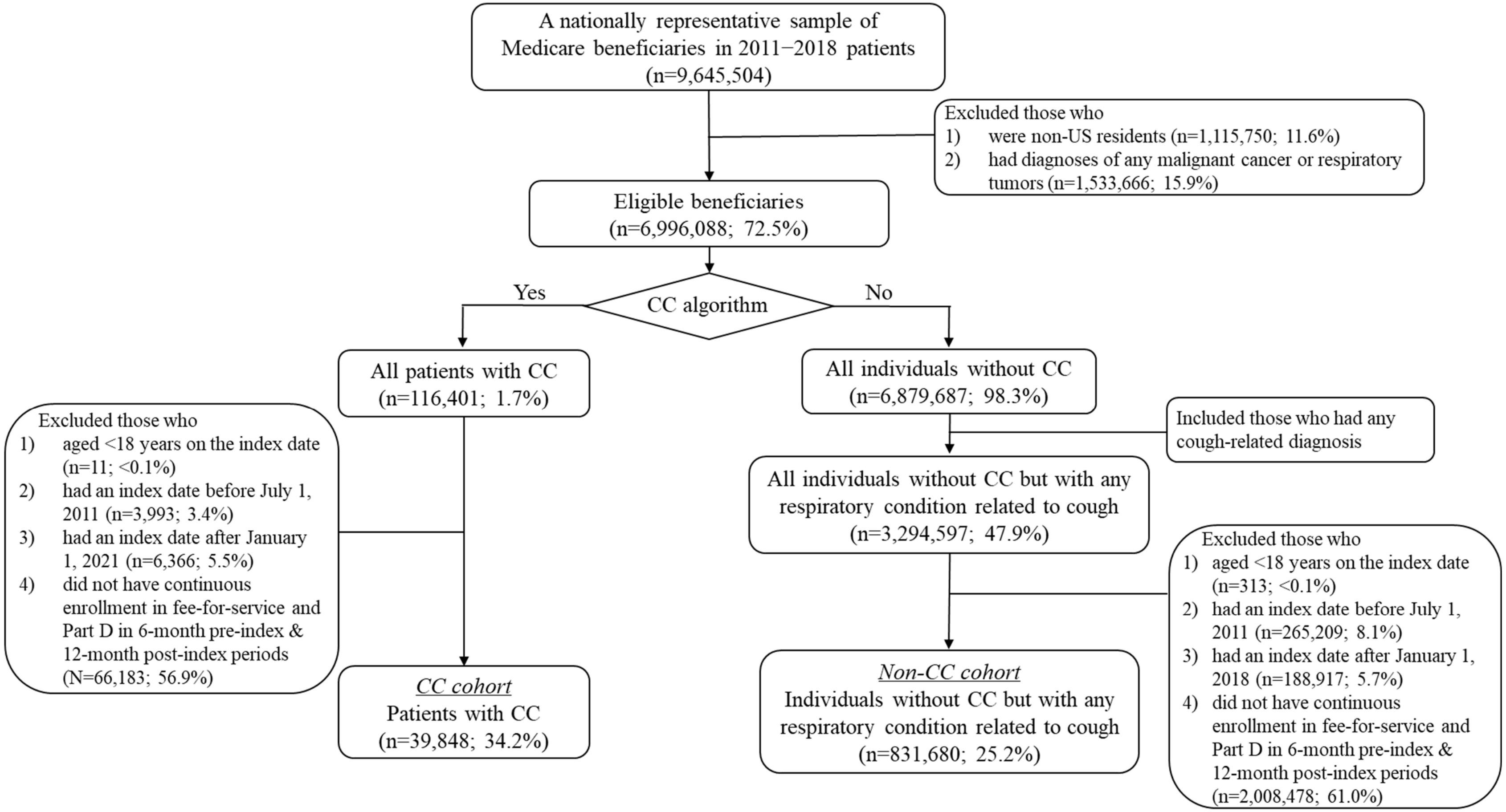

Abbreviations: CC: Chronic Cough.
